# Gender peculiarities of pain syndrome in older age patients

**DOI:** 10.1192/j.eurpsy.2021.1168

**Published:** 2021-08-13

**Authors:** N. Chernus, K. Serdakova, A. Sivkov, R. Gorenkov, S. .Sivkov, T. Savina

**Affiliations:** 1 Department Of Outpatient Therapy, I.M. Sechenov First Moscow State Medical University: Moscow, Russia, Moscow, Russian Federation; 2 Department Of Psychology, I.M. Sechenov First Moscow State Medical University: Moscow, Russia, Moscow, Russian Federation; 3 The Department Of Clinical Pharmacology And Internal Diseases Propaedeutics, the I.M. Sechenov First Moscow State Medical University, Moscow, Russian Federation; 4 Scientific Substantiation Of Health Preservation Of The Population Of The Russian Federation, The N. A. Semashko National Research Institute of Public Health, Moscow, Russian Federation

**Keywords:** pain, gender, vegetative disfunction, alexithymia.

## Abstract

**Introduction:**

The study of gender-related peculiarities of vertebral pain syndrome course in order age patients appears to be highly relevant.

**Objectives:**

Study population included 46 female patients and 38 male patients in the age between 60 and 75 years old; mean age was 67,4±6,6 years old.

**Methods:**

Pain syndrome intensity was assessed using the visual analogue scale (VAS), vegetative disfunction was assessed using A.M. Wayne Questionnaire; the Toronto Alexithymia Scale

**Results:**

The conducted comparative study showed that the male patients perceived the pain syndrome as more intense as compared to the female patients in lumbar spine: 4,5 ±0,8 vs 3,6±0,5 scores (р <0,001) and in thoracic spine: 4,1 ±1,0 vs. 3,4±1,0 (р <0,05). On the other hand, in vegetative dysfunction assessment, the male patients demonstrated generally lower score: 43,3±7,5 scores vs. 59,6+10,3 in female patients, р <0,001. The results of correlation analysis pf interrelations between alexithymia and pain intensity revealed the differences between the study groups in emotion recognition accuracy (Mann-Whitney U-test = 109,00, р = 0.09): female patients showed lower scores (60,7 ±3,5) as compared to the male patients (74,2 ±2,1).

**Conclusions:**

Therefore, the vertebral pain syndrome tends to be more pronounced in older age male patients as compared to the similar population of older age female patients. Therefore, vertebral pain syndrome correction requires multidisciplinary approach, including psychotherapeutic support.

